# Plasma 25-hydroxyvitamin D3 is associated with decreased risk of postmenopausal breast cancer in whites: a nested case–control study in the multiethnic cohort study

**DOI:** 10.1186/1471-2407-14-29

**Published:** 2014-01-17

**Authors:** Yeonju Kim, Adrian A Franke, Yurii B Shvetsov, Lynne R Wilkens, Robert V Cooney, Galina Lurie, Gertraud Maskarinec, Brenda Y Hernandez, Loïc Le Marchand, Brian E Henderson, Laurence N Kolonel, Marc T Goodman

**Affiliations:** 1 Cancer Epidemiology Program, University of Hawaii Cancer Center, 701 Ilalo Street, Honolulu, HI 96813, USA; 2 Cancer Biology Program, University of Hawaii Cancer Center, 1236 Lauhala Street, Honolulu, HI 96813, USA; 3 Department of Public Health Sciences & Epidemiology, University of Hawaii, 1960 East West Road, Biomed D104K, Honolulu, HI 96822, USA; 4 Department of Preventive Medicine, Keck School of Medicine, University of Southern California, 1450 Biggy Street, NRT 1517 J, Los Angeles, CA 90033, USA; 5 Cancer Prevention and Control, Samuel Oschin Comprehensive Cancer Institute, Cedars-Sinai Medical Center, Los Angeles, CA 90048, USA; 6 Community and Population Health Research Institute, Department of Biomedical Sciences, Cedars-Sinai Medical Center, 8700 Beverly Blvd, Room 1S37, Los Angeles, CA 90048, USA

**Keywords:** Breast cancer, 25-Hydroxyvitamin D3, 25-Hydroxyvitamin D2, Ethnic groups, Nested case–control study

## Abstract

**Background:**

Higher sunlight exposure is correlated with lower incidence of breast cancer in ecological studies, but findings from prospective studies regarding the association of circulating levels of vitamin D with the risk of breast cancer have been null. The objective of this study was to examine the relation between plasma levels of vitamin D and the risk of postmenopausal breast cancer.

**Methods:**

We conducted a nested case–control study within the Multiethnic Cohort Study of five race/ethnic groups (white, African-American, Native Hawaiian, Japanese, and Latino) from Hawaii and Los Angeles between 2001 and 2006. Pre-diagnostic plasma levels of 25-hydroxyvitamin D2 [25(OH)D_2_], 25-hydroxyvitamin D3 [25(OH)D_3_] and 25(OH)D (sum of 25(OH)D_2_ and 25(OH)D_3_) were examined among 707 postmenopausal breast cancer cases and matched controls.

**Results:**

Using conditional logistic regression models, 20 ng/mL increases of plasma 25(OH)D_3_ (odds ratio (OR) 0.28; 95% confidence interval (CI) 0.14-0.56) and 25(OH)D (OR 0.43; 95% CI 0.23-0.80) were inversely associated with breast cancer risk among white women, but not among women in other race/ethnic groups. Using two-segmented, piecewise-linear logistic regression models, the change-points of the ORs, either for 25(OH)D_3_ or for 25(OH)D, were detected as 20 ng/mL among whites.

**Conclusions:**

Circulating 25(OH)D_3_ and 25(OH)D were associated with a reduced risk of postmenopausal breast cancer among whites, but not in other ethnic groups, who reside in low latitude regions.

## Background

Ecological studies reporting a correlation between lower solar ultraviolet-B exposure and higher breast cancer incidence and/or mortality [1-3] contributed to the hypothesis that vitamin D may reduce breast cancer risk [4]. Results from retrospective case–control studies are also suggestive of an inverse association of vitamin D intake [5] and circulating 25-hydroxyvitamin D (25(OH)D) [6-8] with the risk of breast cancer. However, results from cohort studies [9-16] and intervention trials [17,18] of the association of circulating vitamin D with breast cancer incidence have been generally null [7,8].

Epidemiological studies of the association of vitamin D with disease are complicated because circulating vitamin D levels are determined by a variety of factors, including ultraviolet radiation exposure, skin sensitivity to sun exposure, and diet [19-22]. Most investigations examining vitamin D and breast cancer risk have been conducted in whites, however; and circulating levels of 25(OH)D, the most reliable indicator of vitamin D status, are known to differ substantially between race/ethnic groups with varying diets and ability to synthesize vitamin D in the skin [19,21,23].

Among several forms of vitamin D, the two major forms are vitamin D3 (cholecalciferol) and vitamin D2 (ergocalciferol). Vitamin D3 is synthesized mostly from the irradiation of 7-hydrocholesterol on the skin and only a few foods naturally contain vitamin D3. In contrast, vitamin D2, at lower circulating levels than vitamin D3, is obtained from intake of foods, fortified products, and supplements. Since skin color is a determinant of synthesis to vitamin D3, it is important to examine the potential modifying influence of race/ethnicity on the relation of circulating vitamin D to disease risk. Specifically, it is unknown whether 25-hydroxyvitamin D2 (25(OH)D_2_) interacts with race/ethnicity on the association of vitamin D with breast cancer risk.

It is plausible that the association of plasma 25-hydroxyvitamin D3 25(OH)D_3_ and 25(OH)D with breast cancer risk is non-linear, and a minimum threshold is needed for vitamin D to exert a protective effect [5]. In this regard, the lower cutoff values that classify vitamin D levels as inadequate or deficient, recently suggested by the Institute of Medicine [24,25] and the Endocrine Society [26], were determined based on previous studies of skeletal health, the results of which may not be directly relevant to breast cancer risk.

We conducted a nested case–control study within the Multiethnic Cohort (MEC) in Hawaii and Los Angeles to test the hypothesis that pre-diagnostic plasma levels of 25(OH)D_2_ and 25(OH)D_3_ are associated with the risk of postmenopausal breast cancer, and that this association varies by race/ethnic group in populations with relatively high year-round levels of sunlight.

## Methods

The MEC is a prospective study of more than 215,000 adults from five race/ethnic groups (white, African-American, Native Hawaiian, Japanese, Latino) in Hawaii and Los Angeles that was established between 1993 and 1996 to examine the association of lifestyle and genes with chronic disease risk [27]. A biospecimen repository was developed within the MEC between 2001 and 2006 including 36,458 postmenopausal women, ages 45–75 years, who agreed to provide blood specimens and a short interview. The MEC was approved by the Institutional Review Boards of the University of Hawaii and the University of Southern California.

Incident invasive breast cancer cases were identified by linkage to the Surveillance, Epidemiology, and End Results Program registries in the states of Hawaii and California through October, 2010, including 729 eligible postmenopausal women with a diagnosis of invasive breast cancer. Controls who were alive and free of breast cancer were randomly selected from the pool of postmenopausal women in the biospecimen repository and matched 1:1 to each case within strata of geographic location (Hawaii or California), race/ethnicity, birth year (±1 y), date of blood draw (±6 mo), time of blood draw (±2 h), hours fasting prior to blood draw (0- < 6, 6- < 8, 8- < 10, and ≥10 h), and hormone replacement therapy use (HRT; as current versus not current). Matched pairs that included a case or control with 25(OH)D_2_ or 25(OH)D_3_ measurements below the limits of detection (10 cases and 10 controls), or outliers (higher levels than three standard deviation (3 cases for 25(OH)D_2_, none for 25(OH)D_3_)), were excluded. After exclusion, 707 matched sets remained for statistical analysis.

Plasma levels of 25(OH)D_2_ and 25(OH)D_3_ were analyzed by isotope dilution liquid chromatography orbitrap mass spectrometry (Laboratory of AAF, ThermoFisher Scientific, Waltham, MA). The assay was validated by the Vitamin D External Quality Assessment Scheme (DEQAS) and National Institute of Standards and Technology (NIST) quality assurance programs. Intra- and inter-assay variability coefficients of variations of 25(OH)D which was the sum of 25(OH)D_2_ and 25(OH)D_3_, were 6.8% and 7.4%, based on the analysis of 47 duplicate and 23 triplicate samples.

Conditional logistic regression with matched sets as strata was used to compute odds ratios (ORs) and 95% confidence intervals (CIs). Levels of 25(OH)D_2_ were coded as a binary variable (0, >0 ng/mL) because the detection level of 25(OH)D_2_ was low. For 25(OH)D_3_ and 25(OH)D, we used continuous variables (10 ng/mL and 20 ng/mL) to facilitate comparison with our results and results from the latest meta-analyses [7,8]. For 25(OH)D, binary variables using several cutoffs (16, 20, and 30 ng/mL) were also used based on the recent definition of vitamin D deficiency or insufficiency [25,26]. Heterogeneity of effect across race/ethnic groups was tested by the Wald test of the cross-product terms for vitamin D and ethnic group. All statistical models were adjusted for potential confounders, including body mass index (<25.0, 25.0-29.9, ≥30.0 kg/m^2^), number of live births (never, 1, 2–3, ≥4, missing), family history of breast cancer (yes, no, missing), use of multivitamin and calcium supplements (yes: taken at least once a week in the last year preceding blood draw; no; missing), season (October to March, April to September), sunburn history (yes, no, missing), and engagement in strenuous sports (never, ≤1 h/week, >1 h/week, missing). The study participants were queried about the frequency and duration of intake of calcium supplements regardless of alone or in combination with vitamin D.

Sensitivity analyses for 25(OH)D_3_ and 25(OH)D were performed among whites by excluding women (by case–control pair) within 6 months, one, two, three, and four years from the date of blood draw. To find the change-point in ORs to identify a threshold effect of 25(OH)D_3_ and 25(OH)D on breast cancer risk, we implemented a two-segmented piecewise-linear logistic regression for each race/ethnic group [28].

## Results

The mean age of the study subjects was 67.8 years (Table 1) and the mean time between the date of blood draw and the date of breast cancer diagnosis was 3.1 years. Compared to controls, in all five race/ethnic groups, cases were likely to be overweight and obese or to have a family history of breast cancer. Dietary and supplementary intake for energy, vitamin D and calcium was not different between cases and controls in every race/ethnic group. Among controls, the mean plasma 25(OH)D_2_ levels were the highest among African-Americans and Japanese, and the lowest among whites. In contrast, mean plasma 25(OH)D_3_ and 25(OH)D levels were the highest among whites and the lowest among African-Americans.

**Table 1:** Basic characteristics of postmenopausal breast cancer cases and controls by race/ethnicity

	**White**				**African-American**				**Native Hawaiian**				**Japanese**				**Latino**			
	**(n = 294)**				**(n = 212)**				**(n = 136)**				**(n = 508)**				**(n = 264)**			
	**Cases**		**Controls**		**Cases**		**Controls**		**Cases**		**Controls**		**Cases**		**Controls**		**Cases**		**Controls**	
	**No.**	**%**	**No.**	**%**	**No.**	**%**	**No.**	**%**	**No.**	**%**	**No.**	**%**	**No.**	**%**	**No.**	**%**	**No.**	**%**	**No.**	**%**
Area																				
Hawaii	137	93.2	137	93.2					68	100.0	68	100.0	215	84.6	215	84.6				
LA	10	6.8	10	6.8	106	100.0	106	100.0					39	15.4	39	15.4	132	100.0	132	100.0
Season																				
Oct-Mar	65	44.2	68	46.3	54	50.9	57	53.8	38	55.9	36	52.9	98	38.6	119	46.8	70	53.0	57	43.2
Apr-Sep	82	55.8	79	53.7	52	49.1	49	46.2	30	44.1	32	47.1	156	61.4	135	53.2	62	47.0	75	56.8
Sunburn history	103	70.1	98	67.1	17	16.2	9	9.0	21	31.3	23	33.8	48	19.0	54	21.4	40	32.3	28	22.8
Family history of breast cancer	22	15.0	13	8.8	21	19.8	22	20.8	12	17.6	10	14.7	38	15.0	28	11.0	15	11.4	8	6.1
Use of multivitamins	82	56.6	73	50.3	59	56.7	56	55.4	36	52.9	33	49.3	135	53.4	126	50.0	63	48.8	69	52.7
Use of calcium supplements	68	47.9	62	43.4	29	29.9	38	36.9	18	26.5	28	43.1	114	45.8	139	55.6	52	42.3	48	38.4
	**Mean**	**SD**	**Mean**	**SD**	**Mean**	**SD**	**Mean**	**SD**	**Mean**	**SD**	**Mean**	**SD**	**Mean**	**SD**	**Mean**	**SD**	**Mean**	**SD**	**Mean**	**SD**
Age	68.5	7.9	68.4	7.7	69.1	7.6	69.2	7.5	65.7	6.8	65.8	6.8	67.8	7.7	67.8	7.7	67.3	6.7	67.3	6.8
Body mass index (kg/m^2^)	25.0	4.5	24.5	5.4	28.0	5.0	28.4	5.7	27.8	5.5	28.1	6.2	24.2	4.2	23.3	3.8	28.2	5.7	27.3	5.6
Live births	2.3	1.6	2.7	1.6	2.9	1.8	2.9	2.0	3.5	2.0	3.5	1.7	2.2	1.4	2.3	1.4	3.5	2.0	4.1	2.0
Sports activity (hour/week)	0.2	0.3	0.2	0.5	0.1	0.3	0.1	0.2	0.2	0.7	0.2	0.4	0.1	0.3	0.1	0.2	0.1	0.4	0.1	0.4
Energy intake, Kcal/day	1,799	649	1,785	714	1,848	1,043	1,834	1,010	2,295	1,191	2,031	1,092	1,814	670	1,900	658	2,189	1,199	2,222	1,369
Vitamin D, IU/1000 Kcal/day	78	44	81	52	74	52	70	52	74	47	81	56	62	38	61	36	72	44	70	58
Calcium, mg/1000 Kcal/day	438	136	436	144	400	146	384	145	361	140	371	170	336	108	331	109	467	142	463	164
Phosphorus, mg/1000 Kcal/day	650	123	658	122	651	137	631	129	578	124	604	139	580	101	568	104	687	127	699	144
Plasma 25(OH)D_2_, ng/mL^a^	3.5	5.4	2.6	4.7	2.2	5.0	3.3	5.5	1.7	3.8	2.8	5.3	4.3	5.7	3.3	4.9	2.5	4.5	3.0	5.4
Plasma 25(OH)D_3_, ng/mL^a^	31.4	10.1	34.8	10.1	23.5	12.3	23.1	9.6	30.9	9.5	30.6	11.6	28.5	9.4	29.3	10.5	25.2	8.1	24.1	8.5
Plasma 25(OH)D, ng/mL^a^	34.9	10.2	37.4	9.7	25.7	12.1	26.4	11.3	32.6	8.7	33.4	12.2	32.7	9.3	32.6	10.2	27.7	9.4	27.1	9.4

*Abbreviation*: *SD* standard deviation, *25(OH)D*_*2*_ 25-hydroxyvitamin D2, *25(OH)D*_*3*_ 25-hydroxyvitamin D3, *25(OH)D* sum of 25(OH)D_2 _and 25(OH)D_3_.

^a^Multiply 2.5 to convert the values to nmol/L.

Plasma concentrations of 25(OH)D_2_ were inversely associated with breast cancer risk among African-American women (>0 ng/mL vs 0 ng/mL: OR 0.29; 95% CI 0.12-0.70), but not among women in other race/ethnic groups (Table 2). A unit increase of 20 ng/mL in plasma concentrations of 25(OH)D_3_ (OR 0.28; 95% CI 0.14-0.56) was inversely associated with postmenopausal breast cancer risk among white women, but not among women in other race/ethnic groups (test for heterogeneity, P = 0.007). Among whites, plasma levels of 25(OH)D considered deficient (<20 ng/ml) led to a 7.5 times greater risk of breast cancer (95% CI 1.41-39.8) compared to women with circulating levels of 25(OH)D at 20 ng/mL or more. For women other than non-Hispanic whites, the association of plasma 25(OH)D levels with breast cancer risk was not statistically significant.

**Table 2:** **Odds ratio (OR) and 95% confidence intervals (CI) for breast cancer by categories in circulating 25(OH)D**_**2**_**, 25(OH)D**_**3**_**, and 25(OH)D levels by race/ethnicity**^**a**^

	**White**				**African-American**				**Native Hawaiian**				**Japanese**				**Latino**				***Test for heterogeneity***^**b**^	
	**(n = 294)**				**(n = 212)**				**(n = 136)**				**(n = 508)**				**(n = 264)**					
**Variables and categories**	**No. cases/controls**	**OR**	**95% CI**		**No. cases/controls**	**OR**	**95% CI**		**No. cases/controls**	**OR**	**95% CI**		**No. cases/controls**	**OR**	**95% CI**		**No. cases/controls**	**OR**	**95% CI**		***Overall***	***Whites vs. others***
**25(OH)D**_**2**_																						
0 ng/mL	80/90	1.00			85/66	1.00			49/43	1.00			124/139	1.00			84/83	1.00				
> 0 ng/mL	67/57	1.29	0.75	2.23	21/40	0.29	0.12	0.70	19/25	0.46	0.16	1.34	130/115	1.32	0.90	1.93	48/49	0.85	0.46	1.56	*P = 0.817*	*P = 0.124*
**25(OH)D**_**3**_																						
10 ng/mL increase	147/147	0.53	0.37	0.75	106/106	1.27	0.91	1.76	118/118	0.89	0.59	1.34	229/229	0.92	0.75	1.12	232/232	1.29	0.87	1.89	*P = 0.059*	*P = 0.007*
20 ng/mL increase	147/147	0.28	0.14	0.56	106/106	1.61	0.83	3.11	118/118	0.80	0.35	1.79	229/229	0.84	0.57	1.24	232/232	1.65	0.76	3.57		
**25(OH)D**																						
10 ng/mL increase	147/147	0.66	0.48	0.90	106/106	1.08	0.79	1.47	118/118	0.79	0.52	1.20	229/229	1.04	0.84	1.28	232/232	1.17	0.84	1.64	*P = 0.086*	*P = 0.051*
20 ng/mL increase	147/147	0.43	0.23	0.80	106/106	1.16	0.63	2.16	118/118	0.63	0.27	1.45	229/229	1.08	0.71	1.65	232/232	1.38	0.71	2.69		
Vitamin D deficiency																						
Cutoff at 16 ng/mL																						
≥16 ng/mL	144/146	1.00			79/85	1.00			65/64	1.00			246/245	1.00			119/119	1.00				
<16 ng/mL	3/1	4.98	0.40	62.1	27/21	1.36	0.59	3.13	3/4	1.44	0.18	11.5	8/9	0.72	0.24	2.14	13/13	1.01	0.31	3.29	*P = 0.395*	*P = 0.465*
Cutoff at 20 ng/mL																						
≥20 ng/mL	136/145	1.00			72/70	1.00			64/61	1.00			233/231	1.00			105/102	1.00				
<20 ng/mL	11/2	7.50	1.41	39.8	34/36	0.74	0.36	1.53	4/7	0.74	0.13	4.29	21/23	0.96	0.47	1.97	27/30	0.90	0.41	1.95	*P = 0.226*	*P = 0.023*
Cutoff at 30 ng/mL																						
≥30 ng/mL	98/115	1.00			29/39	1.00			44/39	1.00			152/144	1.00			45/42	1.00				
<30 ng/mL	49/32	2.56	1.27	5.14	77/67	1.34	0.64	2.79	24/29	0.64	0.28	1.49	102/110	0.86	0.58	1.29	87/90	0.76	0.40	1.46	*P = 0.025*	*P = 0.026*

*Abbreviation*: *25(OH)D*_*2*_ 25-hydroxyvitamin D2, *25(OH)D*_*3*_ 25-hydroxyvitamin D3, *25(OH)D* sum of 25(OH)D_2_ and 25(OH)D_3_.

Note. Values are given in ng/mL. Multiply 2.5 to convert the values to nmol/L.

^a^Modeled through conditional logistic regression after adjustment for season, sunburn history, body mass index, strenuous sports, number of live births, family history of breast cancer, use of multivitamin, and use of calcium supplement.

^b^Tests based on the Wald statistic for cross-product terms between ethnicity and plasma vitamin D level.

Among whites, results from a two-segmented piecewise-linear logistic regression yielded a threshold at 19.4 ng/mL for 25(OH)D_3_ (for unit increase in 25(OH)D_3_, OR 1.11, 95% CI: 0.98-1.25 below the change-point; OR 0.96, 95% CI: 0.92-0.99 above the change-point; test for heterogeneity P = 0.007) and at 19.5 ng/mL for 25(OH)D (for unit increase in 25(OH)D, OR 1.20, 95% CI: 0.97-1.48) below the change-point; OR 0.98, 95% CI: 0.95-1.01 above the change-point; test for heterogeneity P = 0.046) (data not shown). Change-points, either for 25(OH)D_3_ or for 25(OH)D, were not detected among African-American, Native Hawaiian, Japanese, or Latino women.

In sensitivity analyses, no heterogeneity by length of follow-up time after blood draw was detected in the risk estimates by a unit increase of 20 ng/mL of plasma 25(OH)D_3_ or of plasma 25(OH)D concentrations for postmenopausal breast cancer among whites (test for heterogeneity P = 0.939 for 25(OH)D_3_; P = 0.265 for 25(OH)D) (data not shown).

## Discussion

Results from this study add to the growing debate regarding the potential beneficial effects of vitamin D against breast cancer and other malignancies [29,30]. Higher circulating vitamin D3 and vitamin D levels were associated with a lower risk of postmenopausal breast cancer among white women whose circulating 25(OH)D_3_ and 25(OH)D levels exceeded those of women in other race/ethnic groups.

Vitamin D3, which is the major form of vitamin D, is obtained from the diet or through synthesis in the skin from the action of ultraviolet radiation [31]. The inverse association found in this analysis among white women may be explained by the relatively high levels of circulating vitamin D3 achievable among whites living in Los Angeles and Hawaii where ambient levels of ultraviolet radiation are sufficiently elevated throughout the year for adequate cutaneous vitamin D production. White women in our study had higher plasma 25(OH)D levels (means of 34.9 ng/mL in cases and of 37.4 in controls) compared to white women in previous nested case–control studies, with ranges from 20.0 to 31.5 ng/mL among cases and 20.4 to 33.1 ng/mL among controls [9-15,17,18]. This observation is consistent with findings from prospective studies of dietary vitamin D, sun exposure, and breast cancer risk in France [32] and in the United States [33] which showed a lower risk of breast cancer among women who were high dietary vitamin D consumers living in southern regions, but not in northern regions, of these countries.

A meta-analysis of studies measuring plasma 25(OH)D levels and breast cancer risk reported a lower risk of breast cancer associated with a 10 ng/mL increase [7] or 20 ng/mL increase [8] in pooled estimates; however, when the pooled analysis was restricted to prospective studies, this association was null. Our data support the hypothesis that a minimum threshold level of 25(OH)D exceeding 20 ng/ml is necessary for an inverse association with postmenopausal breast cancer risk to be measurable, although the modest number of subjects below this threshold was a concern. A threshold effect rather than a dose-dependent effect of vitamin D with breast cancer risk was recently supported by a re-analysis of data from the Women's Health Initiative [34]: the beneficial effect of vitamin D on reducing breast cancer was examined among women who were not using calcium or vitamin D supplements at baseline, while higher doses of vitamin D did not further decrease breast cancer incidence among women who were using supplements at baseline.

To our knowledge, this is the first case–control study nested in a prospective cohort that examined the association between circulating vitamin D3 and vitamin D (sum of vitamin D2 vitamin D3) concentrations and breast cancer risk in a multiethnic population. Our finding that white women had the highest circulating vitamin D3 and/or vitamin D concentrations is consistent with reports from previous cross-sectional studies [21,23,35-38]. One possible explanation for the unique association of vitamin D with breast cancer risk among white women is a potential modifying effect of this association by vitamin D receptor polymorphisms (*FokI, BgII*) that vary substantially by ethnic group [39-43]. The vitamin D receptor binds the active form of 25(OH)D (i.e., 1,25 dihydroxyvitamin D (1,25(OH)_2_D)) in the nucleus of breast epithelial cells, thereby reducing cell proliferation, and increasing cell differentiation, autophagy and apoptosis [44]. However, it is also possible that light skin pigmentation combined with high sun exposure, rather than differences in vitamin D receptor polymorphisms among whites compared to other women, account for the reduction in breast cancer risk limited to this race/ethnic group [37,45].

In contrast to the results for vitamin D3, we found that a modest reduction in the risk of breast cancer is associated with higher levels of plasma vitamin D2 among African-American women. African-Americans had the lowest plasma 25(OH)D_3_ among the five race/ethnic groups studied. It is possible that the apparent ethnic-specific effect of circulating vitamin D2 on breast cancer risk among African-American women resulted from their relatively low levels of circulating vitamin D3 compared to other race/ethnic groups which may have masked a modest association of risk with vitamin D3. Vitamin D2 (ergocalciferol) is derived from irradiation of plants or yeast and is found in humans when ingested from food or supplements [46]. Recent literature suggests that the binding affinity of vitamin D2 and its metabolites to plasma vitamin D binding protein is weaker than that of vitamin D3 [47,48], but little is known about the physiologic effects or ethnic-specific potency of vitamin D2. Plasma 25(OH)D_2_ levels in our study participants were generally above the assay detection limit, although it is still possible that the association found among white women in our study resulted from chance.

Strengths of this study include its multiethnic composition, the use of pre-diagnosed biologic samples, and a relatively large number of carefully matched cases and controls. High circulating levels of vitamin D in both cases and controls may have attenuated the risk estimates, but study power remained adequate. A further strength of our analysis was the ability to adjust for body mass index, physical activity, as well as calcium and vitamin supplements, factors which are correlated with circulating 25(OH)D levels [49]. Because this information was gathered using a mail survey form, recall may have been imperfect. However, all women included in this analysis were healthy at the time of interview, so any biased responses would be non-differential and likely to attenuate risk estimates toward the null. The number of cases and controls in each ethnic group was not sufficient to stratify the analyses by geographical area and/or by season which is a possible limitation.

## Conclusion

In conclusion, plasma vitamin D was inversely associated with postmenopausal breast cancer risk among white women who reside in latitudes where levels of ultraviolet radiation are comparatively high. It is likely that a minimum threshold of vitamin D exposure from both sun and diet is required to achieve a reduction in breast cancer risk among postmenopausal white women.

## Competing interest

The authors declare that they have no conflict of interest.

## Authors’ contributions

YK participated in the design of the study, performed the statistical analysis, and drafted the manuscript. AAF made substantial contributions to conception and design, acquisition of data, and carried out the laboratory analysis. YBS performed the statistical analysis, contributed to interpretation of data, and helped draft the manuscript. GL made contributions to interpretation of the data. LRW, RVC, GM, BYH, LLM, BEH, LNK, and MTG made substantial contributions to conception and design, acquisition of data, and interpretation of data. MTG also helped draft the manuscript. All authors have been involved in revising the manuscript critically for important intellectual content, and have given final approval of the version to be published.

## Pre-publication history

The pre-publication history for this paper can be accessed here:

http://www.biomedcentral.com/1471-2407/14/29/prepub
